# District-level religious composition and child health in India

**DOI:** 10.1186/s41043-022-00298-7

**Published:** 2022-05-12

**Authors:** Bailey Richards, Krishna Rao, David Bishai

**Affiliations:** 1 Krieger School of Arts and Sciences, Johns Hopkins University, Baltimore, USA; 2 Bloomberg School of Public Health, Johns Hopkins University, Baltimore, USA

## Abstract

**Background:**

Community characteristics are a significant social determinant of child health. Little is known about the effects of social heterogeneity as a specific factor that might impact health. This paper aims to fill the void in research on the health effects of India’s district-level religious heterogeneity.

**Methods:**

Weighted state fixed effects multivariate logistic regression was applied to India’s Third District Level Household Survey (2007–2008). The dependent variables were death of a child under five and indicators of healthcare utilization. The key independent variables were the proportions in the district who were Hindu, Muslim, Christian, Buddhist, and Sikh. The analysis controlled for generic community diversity, household religion, and socioeconomic status. Separate, sub-group analysis focused on Muslims only, Christians only, and Buddhists only.

**Results:**

Multivariate fixed effects models show that a 1% point increase in the proportion of Muslim, Christian, or Buddhist households in a community is associated with respective odds ratios of child death of 1.008, 1.009, and 1.012 of experiencing the death of a child. The impact of a household’s own religious affiliation is statistically insignificant in these models. Higher proportions of Muslims and Christians in a community lower the odds of BCG (a vaccine for childhood tuberculosis) receipt and child healthcare-seeking.

**Conclusions:**

Households residing where there are higher levels of religious minorities in India experience worse child survival. These effects are not mediated by the household’s own religious affiliation. There is evidence that health system performance and quality is systematically worse in communities with higher proportions of religious minorities. Our study can help policymakers identify communities where children may be at higher risk based on community heterogeneity and the potential for insufficient collective action. Policymakers might consider flagging these communities for special attention, because social heterogeneity is likely to be of long duration.

## Introduction

Studies of the association between religion and health in India reveal a paradox: despite, on average, Hindu advantages in socioeconomic status (SES), employment, and education, Muslims and Christians have an average household-level advantage in child health outcomes, even over upper caste Hindus. [3, 4, 11]. Bhalotra reports that during the period from 1960 to 2006 in India, the child mortality rate was 11.29% for Muslims, 13.60% for Hindus overall, and 12.59% for upper caste Hindus. Thus, Muslims had a child survival advantage of 2.31 percentage points over all Hindus and 1.30 percentage points over upper caste Hindus. Some explain this as an advantage accruing mostly to Muslim girls who do not face as much gender discrimination in intra-household resource allocation [3, 4, 11]. Bhalotra [3] also identifies possible causes as closer family ties and healthier unobserved cultural behaviors among Muslims. For example, a study by Geruso and Spears [10] finds that Hindus are 40% more likely to defecate in open areas compared with Muslims. Furthermore, Guillot and Allendorf [12] find that higher urbanization, better water access and sanitation, mother’s work location, and a non-vegetarian diet may contribute to the Muslim advantage.

Menon and McQueeney find a similar survival advantage accruing for Christian children in India, particularly female infants, who have lower mortality and higher height-for-age even compared with upper caste Hindus and Muslims [16]. They attribute their findings to early Christian missionary teachings that emphasized gender equality, education, and sanitary behavior.

However, child health is determined only partially by household or individual decisions. Despite the well-established individual-level Muslim and Christian advantages, little is known about the neighborhood effects on health of living in districts with a higher prevalence of Muslim, Christian, or Buddhist religious minorities. Social and governmental allocations to population-level public health infrastructure also play a part in child mortality. One study shows that increasing the political representation of Muslims can increase the health outcomes in that district [5]. Even as the partition of India resulted in large numbers of Muslims leaving India, there are Muslim majorities within many villages, districts and cities. Among states, Jammu & Kashmir is the only state with a Muslim majority. Christians make up the majority in the northeastern states of Nagaland, Mizoram, and Meghalaya; and Sikhs make up the majority in the Punjab. Although Buddhists are concentrated in Maharashtra, they do not make up the majority in any state. Hindus dominate the rest of India’s 36 states and union territories [20].

Evidence suggests that community characteristics may be a significant determinant of child health. Deshpande [9] investigates district-level determinants of child mortality by state and finds that female literacy, marriage age, percentage of houses with electricity, availability and quantity of health personnel, proportion of scheduled tribes/castes (previously disadvantaged groups that are targeted by affirmative action policies), and population density had the strongest effect. He finds that safe drinking water, proportion living in urban areas, rainfall, and sex distribution were only weakly correlated with child mortality. Bhalotra [2] finds that state health expenditure has an impact on infant mortality only in rural areas after time lags and state-specific trends are included in the model.

A recent study by Munshi et al. [18] reinforces the need for research on India’s health indicators at the sub-national level. They find that state-level child mortality and its rate of change are highly variable between states, despite the country’s overall improvement in these metrics in recent years. As expected, they find that states that have higher health spending per capita, such as Tamil Nadu and Kerala, perform better than lower-health-spending states, such as Bihar and Madhya Pradesh. However, they also identify contributing factors such as poor access to health facilities, degree of healthcare centralization, policy changes, shortage of healthcare workers, and concentration of medical colleges.

Furthermore, Bhattacharya and Chikwama [6] find that significant district-level inequalities in child mortality in India arise from differences in (a) the availability of safe drinking water, (b) female literacy rates, (c) level of development of and equality of access to medical facilities, and (d) social status variables. They also use “percent of female population whose head of household is Muslim” as a regressor for child mortality and find an insignificant effect. However, their study did not account for key factors at the household level, such as education of mother and father.

Disadvantages may accrue in minority communities simply from social heterogeneity that can impede the provision of public goods. Since India is a predominantly Hindu country, districts with a high proportion of religious minorities experience increased religious heterogeneity. When there is a higher-than-average percent Muslim, Christian, or Sikh in a community, a higher-than-average community heterogeneity is observed as well. Heterogeneity can lead to poorer health outcomes due to the inability to achieve political consensus and not due to discrimination or oppression. Alesina et al. [1] report that in the USA more ethnically diverse areas tend to have higher spending, but a lower share of that spending goes toward public goods provision. They theorize that this is due to ethnic distrust between members of different groups. In another study, Cutler and Glaeser [8] find that blacks living in segregated areas or ghettos have significantly worse outcomes than blacks living in racially integrated areas.

It seems that minorities stand to benefit from the integration more so than dominant groups. The positive effects of heterogeneity for minority health are evident globally. Jonsson and Demireva [15] find that local religious diversity, increases odds of self-reported better perception of own health among minorities in the United Kingdom. Likewise, Connor [7] finds that a measure of religious and birthplace diversity called the Theil index, lowers the mortality index by 0.04 for each standard deviation increase in diversity in predominantly Catholic Dublin.

Given that public health allocation decisions across districts are made at state and, occasionally, national levels, districts with a high proportion of Muslims, Christians, or Buddhists might face a disadvantage in resources or staffing. Misallocation may not be intentional. A shortage of health workers with skills or willingness to work in a minority community could make it more difficult to fill vacancies. A systematic tendency of minority groups to live in more remote locations might also lead to statistical confounding. A report commissioned by the Government of India reveals that areas with a higher proportion of Muslims have less access to credit and poorer infrastructure, including water supply facilities, roads, bus stops, sewage, and access to educational institutions. Furthermore, 40% of villages with large Muslim populations did not have access to medical facilities [11].

This paper aims to fill the void in research on the health effects of India’s district-level religious heterogeneity. We use maternal and family health data from 2007 to 2008 on nearly 700,000 households to examine the effects of living in districts with higher proportions of Muslims, Christians, or Buddhists on child health, controlling for household-level religion and other household- and district-level demographic and social factors. Our analysis attempts to disentangle three possible mechanisms for neighborhood religious prevalence to affect child health. The first possibility is shortages or inferior quality of health services in minority communities, and we examine effects of neighborhood religious prevalence on rates of vaccination and healthcare utilization to assess this. The second possibility is that effects of religious prevalence are really just effects of social heterogeneity, and we add Herfindahl–Hirschman Index as a specific measure of social heterogeneity that can control for these effects. The third possibility is that there has been selective migration or historical presence of various religious groups into locations that vary systematically in the amenities necessary for child survival. We add multiple measures of district-level amenities to control for these potentially confounding influences.

## Methods

We use household-level survey data from India’s District Level Household and Facility Survey 3, 2007–2008 [14]. This nationally representative household-level survey had a sample size of nearly 700,000 households. This survey was carried out by the International Institute for Population Sciences in Mumbai on behalf of the Indian Ministry of Health and Family Welfare. Each of the 611 Indian districts in the sample is represented by between 1000 and 1500 households. Our study uses data from 643,944 households where there were ever-married women age 15–49. All variables, including religion, are self-reported and our unit of analysis is the household. Sample households were made up of 76% Hindus, 11% Muslims, 7% Christians, and 6% others [14].

We use sample weighted multilevel logistic regression with state fixed effects to estimate the impact of religious concentration on the health of religious minorities. The analytic sample was composed of 282,784 children who were ever born to DLHS-3-participating households at most 5 years prior to the interview date. To compute respective percentages of households which are Muslim, Christian, Sikh, or Buddhist, which are our independent variable of interest, we aggregate the number of households of each religion and divide by the total number of households within the district. We used Stata/SE 14.2 statistical software to perform the analysis and report odds ratios and t statistics.

The full model with fixed effects is:

$$odds\left( {D_{ijk} } \right) = C + \beta_{1} M_{k} + \beta_{2} X_{jk} + \beta_{3} X_{k} + \beta_{4} HHI_{jk} + \mu_{k} + \varepsilon_{ijk}$$

Subscript “*ijk*” is for “*i*th” child in the “*j*th” household in the “*k*th” district. The dependent variable D_*ijk*_ is a dichotomous indicator of death of a child prior to their 5th birthday. This dummy indicator takes the value of 1 if the child is now deceased and the value of zero otherwise. Only children who were born less than 5 years prior to the interview were included in the analysis. The independent variables include: M_*k*_, which are comprised of indicators for percentage each of Muslim, Christian, Buddhist, and Sikh households in the district; X_*jk*_ which is the vector of household-level determinants of child survival; X_*k*_ which is a vector of district-level determinants of child survival; and Herfindahl–Hirschman (HHI_jk_) which is a measure of district-level diversity. The error term is modeled as a combination of a geographic fixed effect μ_k_ and an individual specific effect ε_ijk_.

Among household-level variables, it was obligatory to control for individual household religion (Muslim, Hindu, Buddhist, Christian, Sikh, and Other) in order to improve the interpretation of district-level proportion of each religion. Hence, dummy variables for each religious affiliation at the household level were included. We also tested a set of interaction terms as the product of own-religion indicator times percent own-religion prevalence in the community.

The rationale for including each of the additional household-specific determinants of child survival was guided by past literature and the theoretical framework of Mosley and Chen [17]. The household-level distal determinants of child survival in the Mosley–Chen framework are maternal autonomy and household wealth, hence our model includes variables for whether mother could read, whether mother worked in the last year, whether the household had health insurance, whether the household lived in a rural area, and the household’s wealth quintile. The DLHS-3 uses households’ reported assets, durables, and amenities to calculate wealth quintiles, which are then compared with the national wealth index, where the first wealth quintile represents the poorest fifth of the population. Another social status distal determinant that has repeatedly shown importance in India has been the caste. Caste hierarchies in India can be extensive and region-specific. Scheduled castes and scheduled tribes are defined by the Indian constitution and represent previously disadvantaged groups that are targeted by affirmative action policies. In the DLHS-3, the simplest depiction of caste status was a variable indicating whether a household was from a non-scheduled caste or tribe. In applying the Mosley–Chen framework to India, researchers have explicitly noted the importance of sex preference as a distal determinant [19]. This variable indicates whether the mother has a stated preference for a male child and may reflect district-level norms that impact health. Since the Mosley–Chen framework includes the household sanitary environment as one of the proximal determinants of child survival, our model includes an indicator for toilet access. “No toilet” is a dummy variable indicating that the family has no access to any type of toilet facility and must practice open defecation. We also include household-level variables for mother’s and father’s years of education. If an individual answered that they had never attended school, his or her years of education were set to zero. Individuals who reported greater than 40 years of education were dropped from the sample, as this group represents outliers. Because our analytical sample was missing less than 0.05% observations relative to the full sample, imputation was not used.

We constructed district-level measures of the proportion of each religion. This model also includes percent of households living in rural areas, because when various religions tend to segregate across urban/rural grounds, this could confound interpretation. We also needed to proxy social norms related to health, as the Mosley-Chen model includes them as its most distal determinants. Thus, we included district average mother’s and father’s years of education, district-level proportions of wealth quintiles, toilet access, mothers’ literacy, no or male sex preference, employment (percent of mothers in district who worked in the last year), health insurance ownership, parents’ schooling; and district-level scheduled caste and scheduled tribe makeup. This allows us to control for community-level effects of these factors on the odds of child death, to ensure that the pure effect of each religion on the relationship is established, and is not confounded with other variables.

In a second model, we include the Herfindahl–Hirschman index (HHI). The HHI is typically used to measure market concentration, but here we use it as the measure of diversity to ensure that the effects of the relative proportions of Muslim, Christian, Sikh, and Buddhist religious minorities are not simply effects of community heterogeneity. We calculate HHI by summing the squares of M_*k*_ for each district. The HHI was scaled to range from a high of 1 when one religion makes up 100% and a low of 0.2 when each of the five major religions accounted for 20% each. In our sample HHI ranged from 0.32 to 0.99 with a mean of 0.77. Hinduism was the most prevalent religion even in the most heterogeneous communities at the bottom decile of HHI. A summary of HHI and other variables is given in Table 1.

**Table 1:** Descriptive statistics on the sample

		Number non-missing	Mean or proportion	(SD)
deadchild2	Child death prior to age 5	282,784	0.04	0.20
bcgvacc	Individual-level received BCG vaccine	147,693	0.82	0.39
poliovacc	Individual‐level received polio vaccine	147,984	0.97	0.16
vaccmotiv1	Relatives friends advised mother to vaccinate last child	282,784	0.22	0.42
openfield	Defecates in open field	281,407	0.64	0.48
sickseek	Sought treatment for sick child	58,995	0.72	0.45
govseek	Sought treatment for sick child at government facility	42,351	0.27	0.44
ultrasound	Individual received ultrasound	282,717	0.01	0.08
anc	Individual received antenatal care	282,774	0.71	0.45
treatsought	Individual sought treatment for pregnancy problem	164,860	0.52	0.50
pmuslim	Percent Muslims in district	278,541	0.15	0.36
phindu	Percent Hindus in district	278,541	0.76	0.43
pchristian	Percent Christian in district	278,541	0.05	0.23
psikh	Percent Sikh in district	278,541	0.02	0.14
pbuddhist	Percent Buddhist in district	278,541	0.01	0.10
nosexpref	Individual‐level no sex preference	108,138	0.40	0.49
boysexpref	Individual‐level boy sex preference	108,087	0.47	0.50
noread	Individual cannot read	173,010	0.82	0.39
healthinsur	Individual has health insurance	279,160	0.03	0.18
wealthquint1	Individual‐level poorest quintile	282,728	0.21	0.41
wealthquint2	Individual‐level wealth quintile 2	282,728	0.22	0.41
wealthquint3	Individual‐level wealth quintile 3	282,728	0.21	0.41
wealthquint4	Individual‐level wealth quintile 4	282,728	0.20	0.40
wealthquint5	Individual‐level least poor quintile	282,728	0.17	0.37
scaste	Individual‐level scheduled caste	282,711	0.19	0.39
stribe	Individual‐level scheduled tribe	282,711	0.18	0.38
nocastetribe	Individual‐level no caste or tribe	282,711	0.40	0.49
notoilet	No toilet in household	282,784	0.63	0.48
rural	Individual‐level rural	282,784	0.82	0.38
stillbirths	Had a stillbirth	282,763	0.04	0.24
spontabort	Had a spontaneous abortion	282,758	0.13	0.46
indabort	Household-level induced abortions	282,767	0.03	0.20
hhi	Herfindahl–Hirschman index of religious heterogeneity	282,784	0.77	0.15
ancvisits	Number of ANC visits at last pregnancy	201,139	5.50	12.58
worklastyear	Individual worked last year	192,785	0.13	0.34
momyearsed ~ t	Mother’s years of education	282,784	3.99	1.80
dadyearsed ~ t	Father’s years of education	282,784	6.44	1.54
scastecompt	District-level % in scheduled caste	282,784	0.17	0.09
stribecompt	District-level % in scheduled tribe	282,784	0.17	0.26
nocastetribe	District-level % in no caste or tribe	282,711	0.40	0.49

To assess the importance of confounding, our analysis started with a simple model featuring only neighborhood religious composition. Analysis sequentially inserted blocks of control variables to assess how much the respective coefficients on proportions of each religion changed as variables were added or dropped.

We repeated the analysis separately for male children’s deaths and for female children’s deaths, to reveal whether religious composition has differential impacts on child survival by sex. This also eliminates the possibility that the observed relationship is attributable to varying-by-religion household-level and community-level social norms promoting preference for a male child. In Table 2, we compare districts with the highest and lowest percentage of Muslim households. In Table 3, we test various models, incorporating household- and district-level variables as well as state fixed effects (Model 4). Table 4 then uses our preferred statistical specification (Model 4) to estimate models stratified by religious sub-group. In order to investigate whether health service utilization might also be affected, we also use the full fixed effects model to test the effect of religious composition on self-reported child receipt of BCG (a vaccine for childhood tuberculosis), polio, and tetanus vaccine (Table 5).

**Table 2:** Comparing descriptive data by districts with lowest and highest percentage of Muslim households

Variable	Districts in lowest quartile			Districts in highest quartile			Z-test or T-test
	Percent Muslim			Percent Muslim			Significance
	Number non-missing	Mean or proportion	SD	Number Non-Missing	Mean or proportion	SD	
Individual-level child death	70,986	0.04	0.19	69,868	0.05	0.22	***
Individual male child death	70,983	0.11	0.31	69,862	0.14	0.35	***
Individual female child death	70,982	0.09	0.29	69,852	0.13	0.34	***
Individual level received BCG vaccine	37,248	0.92	0.27	35,775	0.71	0.45	***
Individual-level received polio vaccine	37,297	0.97	0.17	35,876	0.98	0.15	***
Relatives friends advised her to vaccinate	70,986	0.19	0.40	69,868	0.24	0.43	***
Defecates in open field	70,751	0.59	0.49	69,404	0.58	0.49	
Sought treat for sick child	12,090	0.66	0.47	16,907	0.75	0.43	***
Sought treat for sick child at Govt facility	7,964	0.45	0.50	12,687	0.21	0.41	***
Individual-level ultrasound received	70,982	0.01	0.08	69,842	0.01	0.08	***
Individual received antenatal care	70,986	0.74	0.44	69,868	0.72	0.45	***
Sought treat for pregnancy problems	36,294	0.53	0.50	44,880	0.52	0.50	***
Individual-level Muslim	69,732	0.01	0.11	69,024	0.41	0.49	***
Individual-level Hindu	69,732	0.73	0.44	69,024	0.57	0.49	***
Individual-level Christian	69,732	0.16	0.36	69,024	0.01	0.11	***
Individual-level Sikh	69,732	0.07	0.25	69,024	0.00	0.06	***
Individual-level Buddhist	69,732	0.03	0.16	69,024	0.00	0.07	***
Had an ultrasound	70,982	0.01	0.08	69,842	0.01	0.08	***
Individual-level no sex preference	27,304	0.41	0.49	27,638	0.43	0.50	***
Individual-level boy sex preference	27,304	0.44	0.50	27,630	0.43	0.50	*
Individual cannot read	39,470	0.75	0.44	46,884	0.85	0.35	***
Individual has health insurance	70,259	0.04	0.18	69,129	0.02	0.14	***
Individual received antenatal care	70,986	0.74	0.44	69,868	0.72	0.45	***
Individual-level poorest quintile	70,982	0.22	0.42	69,853	0.21	0.41	***
Individual-level wealth quintile 2	70,982	0.19	0.39	69,853	0.22	0.42	***
Individual-level wealth quintile 3	70,982	0.21	0.40	69,853	0.20	0.40	
Individual-level wealth quintile 4	70,982	0.21	0.41	69,853	0.20	0.40	***
Individual-level least poor quintile	70,982	0.17	0.38	69,853	0.16	0.37	***
Individual-level scheduled caste	70,985	0.18	0.39	69,861	0.16	0.37	***
Individual-level scheduled tribe	70,985	0.37	0.48	69,861	0.08	0.27	***
Individual-level no caste or tribe	70,985	0.27	0.44	69,861	0.44	0.50	***
No toilet or latrine	70,986	0.58	0.49	69,868	0.58	0.49	
Individual-level rural	70,986	0.85	0.35	69,868	0.82	0.38	***
Had a stillbirth	70,975	0.03	0.21	69,861	0.06	0.28	***
Had a miscarriage	70,980	0.11	0.41	69,858	0.15	0.49	***
Had an abortion	70,980	0.02	0.18	69,863	0.03	0.21	***
Herfindahl–Hirschman index of religious heterogeneity	70,986	0.85	0.16	69,868	0.62	0.13	***
Number of ANC visits	52,206	6.23	15.42	50,265	5.38	11.95	***
Mother’s years of education	70,986	4.97	4.75	69,867	3.64	4.64	***
Father’s years of education	70,796	6.74	4.80	69,594	5.74	4.91	***
Individual worked last year	45,514	0.17	0.37	52,254	0.11	0.31	***
Fraction worked last year in district	70,986	0.19	0.15	69,868	0.12	0.14	***
District % no sex preference	70,986	0.56	0.11	69,868	0.56	0.09	***
Fraction Muslim in district	70,986	0.01	0.01	69,868	0.35	0.22	***
Average mothers' yrs school in district	70,986	4.33	1.65	69,868	3.63	2.04	***
Average fathers' yrs school in district	70,986	6.40	1.63	69,868	5.95	1.60	***
Fraction in poorest quintile in district	70,986	0.20	0.20	69,868	0.19	0.16	***
Fraction in 2nd poorest in district	70,986	0.18	0.12	69,868	0.21	0.12	***
Fraction in 3rd poorest in district	70,986	0.20	0.09	69,868	0.20	0.08	***
Fraction in 4th poorest in district	70,986	0.21	0.12	69,868	0.20	0.11	
District level % in scheduled caste	70,986	0.16	0.11	69,868	0.16	0.09	***
District level % in scheduled tribe	70,986	0.35	0.35	69,868	0.08	0.16	***
District level % in no caste or tribe	70,985	0.27	0.44	69,861	0.44	0.50	***
District % no toilet	70,986	0.55	0.31	69,868	0.55	0.32	

* *p* < 0.1, ** *p* < 0.05, *** *p* < 0.01

**Table 3:** Multivariate logistic models of individual odd’s of death in children under 5 in DLHS3

Variable	Model 0	Model 1	Model 2	Model 3	Model 4 (FE)
Percent of Muslim households in district	1.002***	1.001	1.002**	1.001	1.008***
	[3.955]	[1.338]	[2.450]	[1.468]	[4.267]
Percent of Christian households in district	0.992***	0.992***	0.994***	1.005***	1.009***
	[− 10.54]	[− 10.55]	[− 5.046]	[2.980]	[3.473]
Percent of Buddhist households in district	0.983***	0.981***	0.988***	0.997	1.008**
	[− 6.464]	[− 6.893]	[− 4.073]	[− 1.002]	[2.205]
Percent of Sikh households in district	1.001	1.000	1.005***	1.002	0.999
	[1.623]	[0.293]	[3.017]	[0.979]	[− 0.284]
Herfindahl index (HHI)		0.726***	0.697***	0.716***	1.240
		[− 4.456]	[− 4.659]	[− 3.468]	[1.464]
Individual-level Muslim			1.852*	1.910*	1.668
			[1.707]	[1.790]	[1.415]
Individual-level Hindu			1.974*	2.010*	1.752
			[1.890]	[1.939]	[1.557]
Individual-level Christian			2.151**	2.055**	1.818
			[2.104]	[1.975]	[1.630]
Individual-level Sikh			1.875*	1.917*	1.666
			[1.675]	[1.733]	[1.360]
Individual-level Buddhist			1.718	1.734	1.547
			[1.404]	[1.422]	[1.120]
Individual has health insurance			0.873*	0.995	0.971
			[− 1.723]	[− 0.0610]	[− 0.358]
Individual-level mother’s years of education			0.966***	0.982***	0.983***
			[− 10.36]	[− 5.297]	[− 5.074]
Individual-level father’s years of education			0.981***	0.977***	0.978***
			[− 7.094]	[− 8.348]	[− 7.905]
Individual-level poorest quintile			1.377***	1.517***	1.477***
			[5.654]	[6.762]	[6.375]
Individual-level wealth quintile 2			1.297***	1.455***	1.428***
			[4.842]	[6.456]	[6.163]
Individual-level wealth quintile 3			1.242***	1.374***	1.353***
			[4.308]	[5.912]	[5.657]
Individual-level wealth quintile 4			1.219***	1.325***	1.318***
			[4.296]	[5.868]	[5.738]
Individual-level scheduled caste			1.178***	1.135***	1.135***
			[4.810]	[3.566]	[3.540]
Individual-level scheduled tribe			0.988	1.124***	1.119**
			[− 0.305]	[2.611]	[2.443]
No caste or tribe			1.091***	1.025	1.030
			[2.989]	[0.773]	[0.942]
No toilet			1.128***	1.058	1.074*
			[3.742]	[1.530]	[1.942]
Individual-level rural			1.018	1.027	1.032
			[0.513]	[0.724]	[0.845]
Percent in district who cannot read				1.007***	1.019***
				[3.859]	[6.527]
Percent in district who worked in last year				1.003***	1.007***
				[3.175]	[5.278]
Percent in district with no sex preference				0.993**	1.009**
				[− 2.238]	[2.442]
Percent in district with male sex preference				1.002	1.017***
				[0.540]	[4.012]
Percent in district with health insurance				0.988***	0.996
				[− 4.518]	[− 1.430]
Average mother’s years of education in district				0.999***	1.001***
				[− 4.792]	[4.765]
Average mother's years of education in district				1.000*	0.999**
				[1.954]	[− 2.411]
Percent in poorest quintile in district				0.989***	1.003
				[− 4.885]	[0.810]
Percent in wealth quintile 2 in district				0.978***	0.999
				[− 9.395]	[− 0.417]
Percent in wealth quintile 3 in district				1.000	1.007*
				[− 0.105]	[1.903]
Percent in wealth quintile 4 in district				0.973***	1.002
				[− 8.425]	[0.582]
Percent with no access to toilet in district				0.999	0.999
				[− 0.868]	[− 0.759]
Percent in scheduled caste in district				1.007***	1.003
				[3.537]	[1.478]
Percent in scheduled tribe in district				0.996***	0.996**
				[− 3.875]	[− 2.465]
Percent in no caste or tribe				1.003***	0.998
				[3.592]	[− 1.265]
Percent living in rural area in district				1.000	0.997*
				[0.280]	[− 1.787]
Constant	0.0458***	0.0600***	0.0266***	0.0590***	0.000286***
	[− 234.2]	[− 45.81]	[− 9.842]	[− 5.357]	[− 11.43]
Observations	282,784	282,784	274,063	274,063	274,063
Hausman					16.24
Hausman *P*					0

t-statistics in brackets, ***p < 0.01; **p < 0.05; *p < 0.1

Models 3 and 4 also controlled for district-level wealth quintiles, toilet access, mothers’ literacy, no or male sex preference, employment (percent of mothers in district who worked in the last year), health insurance ownership, parents’ schooling; district-level scheduled caste and scheduled tribe makeup; and rurality

**Table 4:** Sub-group models of individual odds of death in children under 5 in DLHS-3

Variable	Odds ratios of fixed effects models of any child death by sub-group					
	Only Muslims	Non-Muslims	Only Christians	Non-Christian	Only Buddhists	Non-Buddhists
Percent of Muslim households in district	1.001	1.008***	0.998	1.008***	0.843**	1.008***
	[0.289]	[3.676]	[− 0.173]	[4.171]	[− 2.048]	[4.317]
Percent of Christian households in district	0.995	1.009***	0.996	1.011***	0.994	1.010***
	[− 0.418]	[3.240]	[− 0.469]	[2.735]	[− 0.103]	[3.711]
Percent of Buddhist households in district	1.015*	1.005	0.987	1.009**	1.033	1.010**
	[1.826]	[1.437]	[− 0.909]	[2.319]	[0.785]	[2.275]
Percent of Sikh households in district	0.959**	1.002	0.924	1.000	1.756	0.999
	[− 2.034]	[0.512]	[− 1.269]	[0.119]	[0.476]	[− 0.341]
Herfindahl index (HHI)	0.656	1.257	1.435	1.301	1.44e−05***	1.277
	[− 1.061]	[1.297]	[0.676]	[1.480]	[− 2.662]	[1.634]
Individual has health insurance	0.755	0.998	0.728	0.980	2.120	0.961
	[− 1.096]	[− 0.0237]	[− 0.801]	[− 0.240]	[1.242]	[− 0.478]
Individual-level mother's years of education	0.983*	0.983***	0.974	0.983***	0.967	0.983***
	[− 1.851]	[− 4.643]	[− 1.527]	[− 4.864]	[− 0.727]	[− 5.038]
Individual-level father's years of education	0.982**	0.977***	0.974*	0.978***	0.974	0.978***
	[− 2.561]	[− 7.766]	[− 1.685]	[− 7.747]	[− 0.686]	[− 7.916]
Individual-level poorest quintile	1.287*	1.509***	1.578	1.475***	1.958	1.478***
	[1.715]	[6.069]	[1.440]	[6.234]	[1.011]	[6.357]
Individual-level wealth quintile 2	1.256*	1.460***	1.250	1.437***	1.116	1.433***
	[1.671]	[5.896]	[0.745]	[6.155]	[0.178]	[6.195]
Individual-level wealth quintile 3	1.130	1.401***	1.370	1.356***	1.528	1.356***
	[0.969]	[5.668]	[1.179]	[5.578]	[0.788]	[5.662]
Individual-level wealth quintile 4	1.301**	1.323***	1.277	1.321***	1.315	1.320***
	[2.405]	[5.201]	[0.952]	[5.678]	[0.549]	[5.746]
Individual-level scheduled caste	1.023	1.136***	0.717	1.145***	568,135***	1.130***
	[0.140]	[3.222]	[− 0.706]	[3.748]	[19.49]	[3.403]
Individual-level scheduled tribe	1.014	1.107**	1.432	1.109**	818,690***	1.118**
	[0.0530]	[2.025]	[0.784]	[2.193]	[37.57]	[2.415]
No caste or tribe	1.103	1.018	0.902	1.034	635,336***	1.028
	[1.426]	[0.485]	[− 0.229]	[1.060]	[19.88]	[0.888]
No toilet	1.186**	1.037	1.231	1.070*	1.604	1.069*
	[2.051]	[0.877]	[1.177]	[1.794]	[1.402]	[1.799]
Individual-level rural	0.959	1.049	0.683**	1.046	0.988	1.034
	[− 0.484]	[1.149]	[− 1.967]	[1.184]	[− 0.0232]	[0.884]
Percent in district who cannot read	1.020**	1.017***	1.034***	1.017***	0.955	1.019***
	[2.271]	[5.518]	[3.191]	[5.432]	[− 0.931]	[6.567]
Percent in district who worked in last year	1.000	1.008***	0.990	1.007***	1.027	1.007***
	[0.0285]	[5.493]	[− 1.065]	[5.499]	[0.959]	[4.986]
Percent in district with no sex preference	0.993	1.012***	1.012	1.011***	1.071	1.009**
	[− 0.635]	[2.846]	[0.648]	[2.821]	[0.921]	[2.500]
Percent in district with male sex preference	1.006	1.018***	0.973	1.020***	1.183*	1.018***
	[0.524]	[3.835]	[− 0.991]	[4.357]	[1.780]	[4.071]
Percent in district with health insurance	1.018*	0.994**	0.992	0.995	1.248*	0.995
	[1.842]	[− 1.997]	[− 0.545]	[− 1.574]	[1.698]	[− 1.514]
Average mother's years of education in district	1.000	1.002***	1.003	1.001***	1.025***	1.001***
	[− 0.321]	[4.669]	[1.568]	[4.636]	[3.553]	[4.655]
Average mother's years of education in district	1.000	0.999**	0.998	0.999**	0.977***	0.999**
	[0.351]	[− 2.507]	[− 0.833]	[− 2.357]	[− 3.116]	[− 2.304]
Percent in poorest quintile in district	1.002	1.000	0.993	1.003	1.076	1.003
	[0.196]	[− 0.0417]	[− 0.373]	[0.819]	[0.649]	[0.844]
Percent in wealth quintile 2 in district	1.015	0.992**	0.966	1.001	1.033	0.999
	[1.486]	[− 2.015]	[− 1.604]	[0.226]	[0.368]	[− 0.220]
Percent in wealth quintile 3 in district	0.982**	1.010***	1.010	1.004	1.033	1.006*
	[− 1.962]	[2.735]	[0.542]	[1.147]	[0.478]	[1.679]
Percent in wealth quintile 4 in district	1.016	0.996	0.992	1.004	1.089	1.003
	[1.421]	[− 0.934]	[− 0.437]	[0.840]	[1.138]	[0.827]
Percent with no access to toilet in district	0.994	1.000	0.996	1.000	1.059*	0.999
	[− 1.595]	[0.00334]	[− 0.502]	[− 0.342]	[1.703]	[− 0.800]
Percent in scheduled caste in district	1.003	1.003	0.988	1.003	0.806***	1.003
	[0.562]	[1.303]	[− 0.666]	[1.504]	[− 2.750]	[1.534]
Percent in scheduled tribe in district	0.990**	0.999	1.001	0.996**	0 .924**	0.997**
	[− 2.471]	[− 0.728]	[0.0803]	[− 2.503]	[− 2.316]	[− 2.231]
Percent in no caste or tribe	0.992**	1.000	0.996	0.999	0.958*	0.998
	[− 2.107]	[0.0493]	[− 0.314]	[− 1.138]	[− 1.806]	[− 1.230]
Percent living in rural area in district	1.006	0.996**	1.010	0.996**	0.962	0.997*
	[1.370]	[− 2.210]	[0.954]	[− 2.075]	[− 1.095]	[− 1.841]
Constant	0.00474***	0.000289***	0.00947*	0.000258***	0.00233	0.000264***
	[− 2.809]	[− 10.97]	[− 1.777]	[− 11.10]	[− 0.648]	[− 11.38]
Observations	41,036	232,411	14,760	258,677	2929	270,634

t statistics in brackets

***p < 0.01; **p < 0.05; *p < 0.1

Models also control for district-level wealth quintiles, toilet access, mothers’ literacy, no or male sex preference, employment (percent of mothers in district who worked in the last year), health insurance ownership, parents’ schooling; district-level scheduled caste and scheduled tribe makeup; and rurality

**Table 5:** Multivariate fixed effects logistic regression models of household health determinants

	Odds ratios from fixed effects logistic models of health determinants				
	Last Child Received BCG	Friends and relatives advised to vaccinate	Defecates in open field	Sought formal care for last child respiratory illness	Sought care at government facility for child’s respiratory illness
*Variables*					
Percent of Muslim households in district	0.991***	1.003***	1.007***	0.998	0.991***
	[− 3.586]	[3.180]	[5.707]	[− 1.163]	[− 3.416]
Percent of Christian households in district	0.993**	1.005***	1.013***	0.994**	0.980***
	[− 2.285]	[3.833]	[5.122]	[− 2.229]	[− 6.152]
Percent of Buddhist households in district	1.003	1.007***	1.005*	0.990**	0.992
	[0.692]	[3.584]	[1.765]	[− 2.325]	[− 1.171]
Percent of Sikh households in district	0.993	1.008***	0.994**	0.995	1.007
	[− 0.946]	[2.855]	[− 1.991]	[− 0.582]	[0.855]
Herfindahl index (HHI)	0.884	1.476***		1.066	1.088
	[− 0.573]	[4.465]		[0.351]	[0.357]
Individual-level Muslim	0.746	1.707***	0.284***	1.239	0.504**
	[− 1.100]	[4.911]	[− 7.028]	[0.631]	[− 2.050]
Individual-level Hindu	1.273	1.212*	0.84	1.061	0.544*
	[0.912]	[1.792]	[− 0.996]	[0.176]	[− 1.842]
Individual-level Christian	1.425	1.191	0.548***	0.982	0.584
	[1.294]	[1.530]	[− 3.269]	[− 0.0525]	[− 1.529]
Individual-level Sikh	2.675***	1.037	0.646**	1.054	0.405**
	[2.700]	[0.290]	[− 2.308]	[0.131]	[− 2.305]
Individual-level Buddhist	1.12	0.972	0.687*	0.82	0.672
	[0.330]	[− 0.217]	[− 1.844]	[− 0.538]	[− 1.021]
Individual has health insurance	1.169	0.895***	0.828***	1.159*	1.048
	[1.607]	[− 3.426]	[− 5.150]	[1.815]	[0.518]
Individual-level mother's years of education	1.077***	1.052***	0.962***	1.016***	0.996
	[19.37]	[29.84]	[− 24.26]	[3.784]	[− 0.671]
Individual-level father's years of education	1.037***	1.010***	0.985***	1.014***	0.995
	[12.89]	[6.493]	[− 9.467]	[4.089]	[− 0.949]
Individual-level poorest quintile	0.479***	1.390***	1,389***	0.585***	2.244***
	[− 11.11]	[10.29]	[133.4]	[− 7.313]	[8.603]
Individual-level wealth quintile 2	0.527***	1.369***	237.0***	0.674***	2.147***
	[− 10.13]	[10.70]	[147.4]	[− 5.717]	[8.973]
Individual-level wealth quintile 3	0.619***	1.287***	55.57***	0.727***	2.213***
	[− 8.158]	[9.521]	[143.3]	[− 5.002]	[10.64]
Individual-level wealth quintile 4	0.673***	1.285***	11.36***	0.888**	1.762***
	[− 7.153]	[11.33]	[104.9]	[− 2.050]	[8.777]
Individual-level scheduled caste	0.858***	1.226***	1.274***	1.036	1.063
	[− 3.675]	[9.538]	[9.553]	[0.781]	[1.056]
Individual-level scheduled tribe	0.781***	1.107***	1.095**	0.783***	1.240***
	[− 4.191]	[3.435]	[2.249]	[− 4.300]	[2.768]
No caste or tribe	0.906***	1.167***	1.201***	0.942	0.956
	[− 2.769]	[8.565]	[8.772]	[− 1.526]	[− 0.903]
No toilet	0.958	1.102***		0.995	0.929
	[− 0.996]	[4.665]		[− 0.0997]	[− 1.342]
Individual-level rural	1.303***	0.958*	3.621***	0.842***	0.927
	[5.334]	[− 1.796]	[43.61]	[− 3.534]	[− 1.291]
Percent in district who cannot read	1.007*	1	1.003	0.998	0.999
	[1.708]	[− 0.113]	[1.386]	[− 0.645]	[− 0.340]
Percent in district who worked in last year	0.999	1.004***	0.996***	0.997*	1.007***
	[− 0.589]	[4.070]	[− 3.099]	[− 1.816]	[3.031]
Percent in district with no sex preference	1.008	1.022***	0.993**	0.991*	0.987**
	[1.458]	[8.003]	[− 1.991]	[− 1.829]	[− 2.143]
Percent in district with male sex preference	1	1.016***	0.991**	0.991*	0.969***
	[0.0421]	[5.051]	[− 2.282]	[− 1.679]	[− 4.370]
Percent in district with health insurance	0.991*	1.008***	1.003	0.994	0.998
	[− 1.858]	[4.317]	[1.221]	[− 1.438]	[− 0.477]
Average mother's years of education in district	1.003***	0.999***	1	1	1
	[8.084]	[− 4.665]	[− 0.379]	[− 0.0898]	[0.631]
Average mother's years of education in district	0.998***	1	1.000**	0.999***	1.001***
	[− 6.797]	[1.445]	[2.240]	[− 4.441]	[2.958]
Percent in poorest quintile in district	1	0.992***	0.940***	0.989***	1.022***
	[− 0.084]	[− 3.285]	[− 19.84]	[− 2.611]	[4.011]
Percent in wealth quintile 2 in district	0.998	0.996*	0.938***	0.995	0.981***
	[− 0.375]	[− 1.651]	[− 17.78]	[− 0.970]	[− 3.282]
Percent in wealth quintile 3 in district	1.007	0.990***	0.956***	0.995	1.029***
	[1.491]	[− 4.128]	[− 14.02]	[− 1.188]	[4.814]
Percent in wealth quintile 4 in district	1.006	1	0.973***	1.007	0.995
	[0.912]	[0.0333]	[− 7.999]	[1.269]	[− 0.859]
Percent with no access to toilet in district	1	1.005***	1.090***	0.995***	0.997
	[− 0.027]	[5.833]	[69.64]	[− 2.897]	[− 1.393]
Percent in scheduled caste in district	1.006*	1.003	0.998	0.998	0.997
	[1.782]	[1.597]	[− 0.723]	[− 0.534]	[− 0.778]
Percent in scheduled tribe in district	1.005**	0.996***	0.998*	1	1.012***
	[2.161]	[− 3.785]	[− 1.740]	[− 0.0998]	[5.107]
Percent in no caste or tribe	1.001	1.002**	0.997**	1.004**	1.001
	[0.510]	[2.268]	[− 2.223]	[2.386]	[0.471]
Percent living in rural area in district	1.003	0.999	0.992***	1.004*	1.005**
	[1.030]	[− 0.588]	[− 5.754]	[1.842]	[2.019]
Constant	16.40***	0.00290***	0.0284***	37.06***	3.151
	[3.047]	[− 15.39]	[− 7.329]	[4.511]	[1.227]
Observations	149,520	697,882	694,272	60,734	43,866

t-statistics in brackets

* *p* < 0.1, ** *p* < 0.05, *** *p* < 0.01

Data from the DLHS-3 have household weights and all regressions adjust for household weights using the “svy” commands in Stata/SE 13 which offer linearized estimates of standard errors that account for the complex survey design.

### Role of the funding source

David Bishai obtained research support from Future Health Systems, a research policy consortium supported by the Department of International development of the United Kingdom. The funding source had no role in the collection, analysis, and interpretation of data; in the writing of the report, and in the decision to submit the paper for publication.

## Results

Table 2 tabulates descriptive data separately for the bottom and top quartile of district-level percentage of Muslims in order to permit insight into the type of confounding that might occur across the spectrum of India’s largest religious minority. We performed this two-way tabulation for all religions in the study, but in the interest of space include only the results for Muslims. In the roughly 70,000 households in districts defined as having the lowest quartile (≤ 1%) of prevalence of Muslims the proportion of child death was 4%. However, in districts at the highest quartile (≥ 32%) of prevalence of Muslims, the odds of child death was 5%. The simple comparative results show that proportion of child death is statistically significantly different across these two quartiles. Table 2 also shows that most control variables are statistically significantly different between the top vs. bottom quartiles. The significant association of religious prevalence with control variables held for other religious groups besides Muslims underscores the need for multivariate regression.

Table 3 shows results from various models. Model 0 only controls for district-level differences in the proportions of Muslim, Christian, Buddhist and Sikh religions. Successive models 1–4 add more controls at the household and community level. Model 4 applies state fixed effects, and according to the Hausman test, it is the preferred model specification. In model 4, an increase in the district-level proportion of the community practicing Islam, Christianity, or Buddhism is a risk factor for child mortality, controlling for the religion of a child’s own household. The household religion is not statistically significantly associated with the odds of child death in the preferred model with state fixed effects. The small size of the effect of the community level religious practice on individual odds of death in children is an artifact of measuring proportion of each religion on a 0 to 100 scale. The coefficients of 1.001 to 1.008 for Percent Muslim in a District are statistically significant in the preferred fixed effects model, and if we interpret coefficients as relative risks, this implies that a one-percent increase in a district’s percentage of Muslim population increases the relative risk of a child death by between 1/1000 and 8/1000. Since the proportion of Muslims in a district ranged from 0 to 99.6 in the sample, an increase from 0 to 100% in Muslim population would increase the relative risk of child death by 10–80%. Similar interpretations would apply to the coefficients shown in Table 3 for percentages of Christians, Buddhists, and Sikhs in a district.

The various models in Table 3 apply increasingly extensive controls for confounding. Model 1 introduces the Herfindahl Index to help distinguish pure effects of the existence of multiple different religions (of any type) from the effects of increases in the proportion of any particular religion. Religious homogeneity/monopoly was found to be an environmental factor predicting lower odds of child death in models 1–3, but not in the preferred model 4. Note, that the HHI is measured on a 0–1 scale with 0 being the most heterogeneous religious makeup of a community and 1 implying a religious monopoly. The coefficients on HHI range between 0.726 (*p* < 0.01) to an insignificant 1.240, implying that more homogeneity signified by a higher HHI lowers the odds of child death, but some of the HHI effect was confounded by community and household characteristics, because as these variables were added in Models 2, 3, and 4 the coefficient on HHI declined.

As shown in Table 3, Model 2 adds household-level control variables, Model 3 adds district-level control variables, and Model 4 adds state dummy variables to purge state-level fixed effects. Model 4 in the same table shows that—after controlling for pure effects of (a) religious heterogeneity in a community; (b) religion practiced in a home; and (c) community wealth and household wealth—living in a community with higher proportions of Muslims or Christians, or Buddhists is associated with higher odds of child death. Percentage of Sikhs has no statistically significant association with the odds of child death, but fewer than 10% of districts in India have more than 1% of Sikh practitioners, so the precision on this estimate may be lower. Table 3 shows that there were state-level fixed effects that were biasing the Model 3 coefficients on each religion’s proportion toward the null. Furthermore, all of the effects of the household-level religious affiliation that were significant in models 2 and 3 appear to have been biased due to fixed effects, and Model 4 shows that there were no statistically significant differences in the odds of child death related to the religious practice of the individuals in a household. Thus, our results do not support efforts to explain community effects of religion on health as due to cultural practices inside households that are associated with religious affiliation. Religious prevalence is more likely to be associated with child death rates via effects mediated outside of households.

The odds ratios from the other independent variables shown in Table 3 were consistent with expectations. We found that variables such as belonging to a scheduled caste or tribe (*p* < 0.01), no toilet (*p* < 0.10), and membership in lower wealth quintiles 1–4 (*p* < 0.01), all had a harmful effect and statistically significantly increased the odds of losing a child.

On the other hand, among household-level characteristics, only mother’s and father’s years of education prove to be significantly protective against the risk of child death at the 99% confidence level. Education has a protective effect, where each additional year of a mother or father’s education reduces the odds of death by 2% after we account for all household-, district-, and state-level effects in Model 4.

In separate analysis (not shown), we analyzed the connection of the odds of child death to district religion by child sex and found that living in a community with higher proportions of Muslims or Christians, or Buddhists has nearly the same adverse effect on the odds of death of both female and male children. For instance, higher percentage of Muslims, Christians, or Buddhists in a district increase the odds death in girls to 1.006, 1.009, and 1.012, respectively, and the odds of death in boys to 1.007, 1.009, and 1.011, respectively (all results with *p* value > 0.01).

Table 4 uses our preferred statistical specification (Model 4) to estimate models stratified by religious sub-group. These estimates measure the effect of the proportion of Muslim households in a district on the odds of child death of only households that were Muslim and compares it to the effect on only households that were not Muslim with analogous measures for Christians and Buddhists. The sample of Sikhs was too small to support this analysis. The effect of the variable “Percent of Muslim Households in a District” on the odds of child death among only Muslim households was not statistically significant, while its effect on the odds of child death among households that were not Muslim was 1.008 (*p* < 0.01). The effect of the variable “Percent of Christian in a District” on the odds of child death among only Christian households was not statistically significant, while its effect on the odds of child death among households that were not Christian was 1.011 (*p* < 0.05). The effect of the variable “Percent of Buddhist Households in a District” on the odds of child death among only Buddhist households was not statistically significant, while its effect on the odds of child death among households that were not Buddhists was 1.010 (*p* < 0.05). These results show that the effect of a higher percentage of any minority religion in a district appears to have no statistically significant effect on odds of death of children whose household practices that same particular religion. However, a higher percentage of the population practicing a minority religion in a district increases the odds of child death among households belonging to other religious groups. Thus, these results are consistent with a theory that the mechanism for the mortality effect in children is not driven by being in a household whose religious practices are being reinforced by community members with similar beliefs and behaviors.

Table 5 attempts to determine whether the effects of each religion’s proportions in a district are associated with health system determinants of child survival. It shows that children are less likely to receive a BCG (a vaccine for childhood tuberculosis) as the proportion of Muslim or Christian households increases in a district (*p* < 0.01). Table 5 shows that these reductions in the vaccine uptake are unlikely to be social effects of vaccine resistance in minority groups because increases in the proportion Muslim, Christian, Buddhist, and Sikh all increase the odds that a woman will be advised by friends and relatives to vaccinate her child. Higher percentage of either Muslim, Christian, or Buddhist households in a district is associated higher odds of defecating in an open field rather than a toilet or latrine. Increases in the percentage of Muslim or Christian households in a district also lower the odds of seeking care at a government facility among the 43,866 households reporting a respiratory illness in the last 2 weeks.

## Discussion

Our results indicate that a 1% increase in the percentage of Muslim households in a community is associated with a 1.008 (*p* < 0.01) increased odds of child death, when controlling for household- and district-level effects, as well as state fixed effects. These are actually not small effect sizes, because percentage of Muslim households in the community ranges from 0 to 0.99. Holding everything else equal, moving from a district with no Muslims to one with 100% Muslims would increase the risk of child death by 80%. Odds ratios of child death from a 1-percent increase in Christians or a 1-percent increase in Buddhists were elevated at 1.009 (*p* < 0.01) and 1.008 (*p* < 0.01) respectively.

There were some limitations of the study. All of the variables are self-reported, introducing the possibility of reporter bias. However, there is no reason to believe that this reporting bias should systematically affect predominately Muslim, Christian, or Buddhist districts differently. Although the results are consistent across all four models, we are unable to establish the temporal sequence or direction of causality. In other words, it is possible that Muslims, Christians, or Hindus tend to locate in districts that already do poorly in terms of the elevated risk of child death. A stronger study design would have included multiple waves of data in order to assess how changes in religious proportions affect mortality. Additionally, our dataset does not indicate child age at death, and we rely instead on household reports of whether a child who was less than five had died. This limits the comparability of our analysis to survival studies where child’s age at death is analyzed. However, our analysis of the log odds of child death prior to age 5 would be broadly comparable to the numerous studies of determinants of the under-5 mortality ratio.

We identify four possible mechanisms driving this relationship between the district-level religious heterogeneity and the risk of child death. First, it is possible that predominantly Hindu Indian states unintentionally or intentionally allocate fewer health resources to Muslim, Christian, and Buddhist districts. This does not necessarily not indicate outright structural discrimination. Differential health resource supply may be due to fundamental shortages in the numbers of health workers from religious minorities. Hindu health workers might have a preference to work in Hindu districts. In testing this explanation, we estimated effects of district religious proportion of Muslims, Christians, and Buddhists on lowering BCG vaccination rates in children, as well as care seeking for sick children (Table 5). These health system performance and access indicators are far from definitive and a comprehensive comparison of the availability and quality of health services in religious minority communities will require further research.

A second explanation appears less likely and would hold that Muslim, Christian, and Buddhist communities practice behaviors that increase the odds of child death. This is unlikely, because Model 4 in Table 3 shows no statistically significant effect of a household’s religion on child survival in models that control for state fixed effects. Furthermore, higher percentage of Muslim, Christian, or Buddhist households in the community increases the odds that parents were encouraged by family members and friends to give vaccinations to children. Future research should identify and explore the effects of alternative social norms and community-level behaviors in such districts.

Our evidence argues against a third explanation that the effects of increases in religious minority prevalence in a district is not an effect of that religion per se so much as an effect of non-specific community heterogeneity. Prior work had shown the community diversity can reduce the consensus to pursue social objectives. Including a Herfindahl index to control for nonspecific heterogeneity actually made the effect sizes of specific religions bigger. As expected, community religious homogeneity, as indicated by a higher Herfindahl index, was a protective factor for child survival, and greater religious monopoly (higher HHI) was associated with lower odds of child death.

A final explanation that minority status is inherently stressful is also not fully consistent with the data. Table 5 showed that the coefficient on the percentage of Muslims in a district has no significant association with the risk of child death in Muslim households but it is significant in non-Muslim households. If the stress of being a minority is the mechanism, then the religious minorities should have seen protective benefits when the community prevalence of their religion increased. Minority stresses would not explain why the non-Muslim households experienced a higher risk of child death when the proportion of Muslims in a district increased.

Among the theories we considered, the best explanation of these patterns could be that the Indian health system was not successfully addressing disparities in the availability and quality of the public health and clinical services environment for districts with higher proportions of religious minorities. However, we may not have been exhaustive in thinking of other possible ways to account for our findings.

## Conclusion

The results of this study show that communities with higher prevalence of any of the three leading religious minorities in India—Muslims, Christians, and Buddhists—may be experiencing disparities in child survival. Given the large size of India’s religious minority groups, these results are concerning. India’s Muslim population is projected to grow much faster than its Hindu population, and by 2050 the country is expected to have the largest population of Muslims in the world [13]. It is increasingly crucial that Indian health policy makers attend to religious disparities in health. Our study provides further evidence pointing to a need for regulators to attend to potential gaps in health service coverage in communities to prevent further marginalization of religious minorities in Indian society.

## Data Availability

All data used are publicly available. A Stata file is available.
